# Pap testing among newly diagnosed women living with HIV/AIDS (WLWHA) in South Carolina (SC): routine screening and abnormal follow-up behaviors of HIV-positive female SC medicaid recipients 18-64 years between 2005-2009

**DOI:** 10.1186/1750-9378-7-S1-P18

**Published:** 2012-04-19

**Authors:** Lisa T Wigfall, Heather M Brandt, Wayne A Duffus, Sharon M Bond, Robin Puett, Heather Kirby, Saundra H Glover, James R Hébert

**Affiliations:** 1 University of South Carolina (USC) – Arnold School of Public Health, (a) Department of Health Services, Policy and Management, (b) Department of Health Promotion, Education, and Behavior, (c) Department of Epidemiology and Biostatistics, Columbia, SC, USA; 2 University of South Carolina – School of Medicine, Division of Infectious Diseases, Columbia, SC, USA; 3 South Carolina Department of Health and Environmental Control (SCDHEC), HIV/STD Division, Columbia, SC, USA; 4 Medical University of South Carolina – College of Nursing, Charleston, SC, USA; 5 University of Maryland – School of Public Health, College Park, MD, USA; 6 South Carolina State Budget and Control Board, Office of Research and Statistics, Columbia, SC, USA

## Background

Oncogenic human papillomavirus (HPV) infection is a main cause of cervical cancer. HIV-positive women are at an increased risk of becoming infected with high risk HPV (hrHPV) types that can lead to cervical cancer. Unlike other AIDS defining cancers, such as Kaposi’s sarcoma and Hodgkin’s lymphoma, the incidence of invasive cervical cancer among WLWHA has not decreased with the arrival of highly active antiretroviral therapies (HAART).[1,2] Annual Pap tests are recommended for WLWHA because the risk of developing cervical cancer, an AIDS defining illness, is increased in this group.[3] Early detection of abnormal, precancerous cells and following up on abnormal cytology results is essential to the prevention and control of cervical cancer among WLHWA.

## Material and methods

Approval was obtained from USC and SCDHEC’s institutional review boards (IRBs) to link the SC Medicaid and HIV/AIDS Reporting System (HARS) databases. A purposive sample of 1,183 HIV-positive females 18-64 years old who had an initial HIV-positive diagnosis, and were enrolled in the SC Medicaid database between January 1, 2005 and December 31, 2009 was obtained. Routine Pap testing and abnormal cytology follow-up behaviors are described. Frequencies and proportions are reported.

## Results

Among our sample of WLWHA, 18% (216 of 1,183) had a Pap test during the same year of their initial HIV-positive diagnosis. Over the 5-year period, 45% (532 of 1,183) did not have a Pap test. An abnormal Pap test diagnosis was found in 21% (250 of 1,183) of our sample. Of the 250 WLWHA who had an abnormal Pap test diagnosis between 2005-2009, 42% (105 of 250) had a follow-up Pap test within one year. Only 34% (36 of 105) of those who had a follow-up Pap test within the past year did so within four months of the initial abnormal Pap test diagnosis.

## Conclusions

Adherence to recommended cervical cancer screening guidelines among our sample of WLWHA was suboptimal. Our study’s findings highlight failures across the cancer care continuum despite the increased prevalence, incidence, and persistence of hrHPV infection among WLWHA. These data are especially alarming given challenges with linking and retaining newly diagnosed WLWHA into HIV treatment care. Prevention and control efforts are needed to improve adherence to cervical cancer screening among this high risk group of WLWHA. This includes multi-level interventions that address system-level factors, as well as patient-provider communication. It is our recommendation that these prevention and control efforts target providers and WLWHA independently.

## References

[B1] Palefsky J Curr Opin HIV AIDS 2009 4 1 52 56 10.1097/COH.0b013e32831a7246 19339939 PMC2756707

[B2] Heard I Curr Opin HIV AIDS 2009 4 1 68 73 10.1097/COH.0b013e328319bcbe 19339941

[B3] Chaturvedi AK JNCI 2009 101 1120 1130 19648510 10.1093/jnci/djp205 PMC2728745

